# Loose Ends in the *Cortinarius* Phylogeny: Five New Myxotelamonoid Species Indicate a High Diversity of These Ectomycorrhizal Fungi with South American *Nothofagaceae*

**DOI:** 10.3390/life11050420

**Published:** 2021-05-05

**Authors:** María Eugenia Salgado Salomón, Carolina Barroetaveña, Tuula Niskanen, Kare Liimatainen, Matthew E. Smith, Ursula Peintner

**Affiliations:** 1 Centro Forestal CIEFAP, CC 14, Ruta N° 259 km 16.24, Esquel 9200, Argentina; cbarroetavena@ciefap.org.ar; 2 Universidad Nacional de la Patagonia S.J. Bosco, Esquel Sarmiento 849, Chubut 9200, Argentina; 3 Consejo Nacional de Investigaciones Científicas y Técnicas (CONICET), Godoy Cruz 2290, Buenos Aires 1425, Argentina; 4 Jodrell Laboratory, Royal Botanic Gardens, Kew, Surrey TW9 3AB, UK; tuula.niskanen@cortinarius.fi (T.N.); k.liimatainen@kew.org (K.L.); 5 Department of Plant Pathology, University of Florida, P.O. Box 110680, Gainesville, FL 32611, USA; trufflesmith@ufl.edu; 6 Institute of Microbiology, University Innsbruck, 6020 Innsbruck, Austria; Ursula.Peintner@uibk.ac

**Keywords:** Telamonia, Chile, Argentina, hidden diversity, *C. egonii*, *C. neuquensis*, *C. gracilentus*, *C. pseudoxiphidipus*, *C. voluptatis*

## Abstract

This paper is a contribution to the current knowledge of taxonomy, ecology and distribution of South American *Cortinarius* (Pers.) Gray. *Cortinarius* is among the most widely distributed and species-rich basidiomycete genera occurring with South American *Nothofagaceae* and species are found in many distinct habitats, including shrublands and forests. Due to their ectomycorrhizal role, *Cortinarius* species are critical for nutrient cycling in forests, especially at higher latitudes. Some species have also been reported as edible fungi with high nutritional quality. Our aim is to unravel the taxonomy of selected *Cortinarius* belonging to phlegmacioid and myxotelamonioid species based on morphological and molecular data. After widely sampling *Cortinarius* specimens in Patagonian *Nothofagaceae* forests and comparing them to reference collections (including holotypes), we propose five new species of *Cortinarius* in this work. Phylogenetic analyses of concatenated rDNA ITS-LSU and *RPB*1 sequences failed to place these new species into known *Cortinarius* sections or lineages. These findings highlight our knowledge gaps regarding the fungal diversity of South American *Nothofagaceae* forests. Due to the high diversity of endemic Patagonian taxa, it is clear that the South American *Cortinarius* diversity needs to be discovered and described in order to understand the evolutionary history of *Cortinarius* on a global scale.

## 1. Introduction

*Cortinarius* (Pers.) Gray is the most species-rich ectomycorrhizal genus in South American *Nothofagaceae* forests [1]. Due to their ectomycorrhizal role, *Cortinarius* species are critical for nutrient cycling in forests, especially at higher latitudes [2]. Additionally, some *Cortinarius* species, including *C. magellanicus* Speg. complex, *C. xiphidipus* Moser and Horak, *C. austroturmalis* Moser and Horak, *C. pugionipes* Moser and Horak, *C. effundens* Moser and Horak, *C. cervinus* Moser and Horak or *C. lebre* Garrido, have been reported to be edible and of high nutritional quality [3,4,5,6]. Several species are also renowned for their antioxidant, antimicrobial and acidifying properties [3,7].

Even though several authors have contributed to the knowledge of *Cortinarius* associated with South American *Nothofagaceae* forests ([5,8,9,10,11,12,13,14,15,16,17,18,19,20,21,22], among others), the biodiversity of this genus in the Southern Hemisphere is incredibly high and remains insufficiently studied [23,24]. Recent studies showed, for example, that *C. magellanicus*, which was previously reported as widely distributed and shared among various *Nothofagaceae* hosts in the Southern Hemisphere [5,6,25,26,27,28,29], is a complex of species. The *C. magellanicus* group is composed of at least four phylogenetic lineages, each with strong regionalism and distinct host associations [15].

Phylogenetic analyses have helped to delimit taxonomic entities within *Cortinarius* [16,23,30,31,32,33,34,35,36,37,38,39] and have shown that many morphologically delimited subgenera and/or sections represent unnatural groups [23,24,31,37,40,41,42,43]. In particular, section *Myxotelamonia* was proposed by Moser et al. [5] (p. 22) for endemic telamonioid *Cortinarius* species from South America. These telamonioid taxa all have a clearly gelatinized epicutis with grayish tints in the lamellae but they are from different subgenera [24] (p. 1049). This paper is a contribution to the current knowledge of South American *Cortinarius* taxonomy, ecology, and distribution. The aim of this paper is to describe five new *Cortinarius* species based on morphological and molecular data. Moreover, we tried to place these myxotelamonioid species into *Cortinarius* sections or phylogenetic lineages, but we were not successful due to the lack of closely related reference sequences. As a first step, the South American *Cortinarius* diversity needs to be discovered and described. We regard these South American taxa as essential puzzle pieces that are necessary for understanding the evolutionary history of *Cortinarius* on a global scale. 

## 2. Materials and Methods

### 2.1. Field Work

Samples of *Cortinarius* specimens were collected in *Nothofagaceae* spp. forests in Argentina and Chile during three consecutive mushroom seasons (2015-2017) (Table 1). Study sites were in NW Patagonia of Argentina and Chile, in habitats of the Sub-Antarctic Province, Sub-Antarctic Domain [44]. 

### 2.2. Morphological Study

Macroscopic descriptions were made from fresh basidiomata. Colours of the basidiomes were documented with a color code [45]. UV recordings were made on fresh and dried (exsiccatae) basidiomata using a 366 nm UV lamp. KOH reactions, where relevant, were made on dried basidiomata [46]. Microscopic data were documented with a Nikon camera D70 in combination with the computer program LASX (https://www.leica-microsystems.com 1 April 2017) and ImageJ (https://imagej.nih.gov/ accessed on 05 May 2021). Microscopic characteristics are from dried specimens (exsiccatae) revived in 3% KOH, sulpho-vanillin, Melzer’s reagent or cotton blue following [47] (pp. 43–114). The possible dextrinoid reaction of basidiospores was observed from pieces of lamellae placed in Melzer’s reagent for five minutes. Basidiospore measurements (n≥ 80) were made in 3% KOH from basidiospores taken from the spore deposits from the apex of the stipe and veil tissue. For statistical evaluation, 125 to 150 spores were measured. Spore measurements are given as (min) mean ± standard deviation (max). Studied material is deposited in the IB, HCFC and CORD herbaria (Table 1). 

### 2.3. DNA Extraction, PCR Amplification and Sequencing

To establish phylogenetic relationships, ITS-rDNA sequences were produced as previously described [44] using the primers ITS1 and ITS4 [48]. The rDNA LSU region was amplified with the primer combination LR0R and LR05 [48]. PCR amplifications of RPB1 domains A-C were made with the primer combination RPB1-A and RPB1-C [49]. Sequences were assembled and edited with Sequencher 4.1 (Gene Codes, Ann Arbor, Mich., USA). As a first step, a Blast search was conducted in UNITE (https://unite.ut.ee 1 April 2017). Sequences of closely related *Cortinarius* species were then downloaded from GenBank (http://ncbi.nlm.nih.gov 1 April 2017) and UNITE. ITS sequences from several types of specimens were also included in the study (Table 1). A total of 28 ITS sequences from the five new species were produced for this study. In addition, eleven LSU sequences and five RPB1 sequences were generated. The newly generated sequences were submitted to GenBank under the accession numbers MN707570–90, MT925622–25; MT925951–53, MW546828–32.

### 2.4. Data Analysis

Data analysis was carried out in a two-step process: A first analysis was based on ITS rDNA sequences only. The best reference sequence database is available for this DNA barcoding region, including sequences from several Patagonian holotypes. As a second step, we aimed at placing our terminal clades into known *Cortinarius* sections or lineages based on concatenated ITS-LSU and ITS-LSU-RPB1 sequences. This was done separately due to the fact that different reference sequences were available for the LSU and RPB1 markers. 

A total of 64 rDNA ITS sequences were aligned and manually adjusted in MEGA X [50]. Reference sequences were selected and downloaded for closely relates species based on morphology or based on sequence similarity resulting from BLAST analyses. The evolutionary history was inferred by using the Maximum Likelihood method based on the Hasegawa–Kishino–Yano model + G, parameter = 0.2332. All positions with less than 95% site coverage were eliminated. There was a total of 560 positions in the final dataset.

To evaluate the robustness of the branches in the phylogenetic trees, parsimony-based bootstrap analyses were applied. The bootstrap analyses were conducted using 1000 replications, an SPR search method, and search level 5. The alignment is composed of 653 nucleotides (including gaps). Bayesian Posterior Probabilities were calculated with Mr Bayes 3.2. [51]. Bayesian analysis was carried out with two independent four-chain runs, sampling over 2 million generations.

In addition, two combined phylogenetic analyses were carried out: the first was based on rDNA data only (ITS and LSU), and the second on a combined dataset containing combined sequences spanning RPB1 regions, the ITS regions, and about 600 bases of the 5′-terminal large subunit (LSU) domain (D1/D2). The alignment of the combined ITS and LSU data contained 1328 positions, and 52 taxa. ML analysis was carried out based on the best model (GTR + G parameter = 0.2131) and 1164 positions were analyzed. The tree with highest log likelihood was −10619.28. The alignment of the combined RPB1, ITS, and LSU sequences contained 99 sequences and 2773 positions after the exclusion of ambiguous regions. ML analysis was carried out based on the best model (Tamura 3-parameter + G, parameter = 0.2462), and 1697 positions were analyzed. Two separate Mr Bayes runs were run under the general time-reversible model with gamma-distributed rate variation. Runs included four incrementally heated chains that were run for 10 million generations each, sampling every 100th generation and with the first 2.5 million generations discarded as burn-in. For further evaluation of branch robustness, parsimony-based bootstrap analyses were applied as described above, with 1000 replications.

Statistical analyses were performed with the width, length, and volume of spores. The width, length, and volume of spores between the species did not meet the assumptions of normal distribution and equal variances using the Shapiro-Wilk and Levene tests [52]. Therefore, differences in the width, length, and volume of spores between species were analyzed using non-parametric Kruskal-Wallis ANOVAs performed at the 0.05 significance level, using the statistical package InfoStat for Windows version 2017 [53]. Test for normal distribution and QQ-Plots were performed with R package (R Core Team 2019).

## 3. Results

### 3.1. Molecular Data

**Figure 1:**
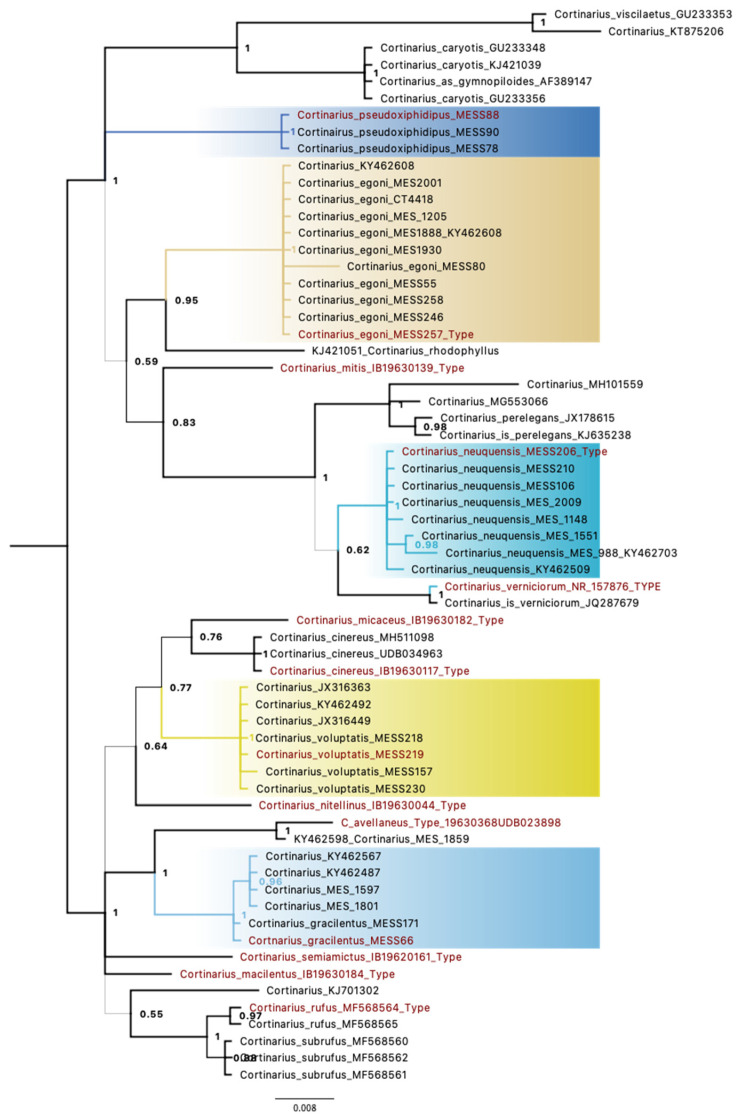
Phylogenetic relationships of the five new species of *Cortinarius* from South American *Nothofagaceae* forests based on rDNA ITS sequences. Bayesian posterior probabilities are provided beside each node. Sequences generated from type material are highlighted in red.

The ITS-based phylogeny with the best ML log likelihood -2174.22 allowed for the best comparison to available reference sequences, including sequences generated from type specimens. All five species described here form well-supported clades (Bayesian posterior probability(BPP) > 0.99, Bootstrap Score (BS) > 95%) in the ITS phylogeny (Figure 1). The sister-group relationships are well-resolved in *C. gracilentus* only, where closely related reference sequences of *C. avellaneus* are available. *Cortinarius egonii* is sister to *C. rhodophyllus* Moser & Horak (BPP = 0.95), but only one reference sequence is available for *C. rhodophyllus,* but it was not obtained from type material. The ITS sequence generated from the type of section *Myxotelamonia*, *C. cinereobrunneus* IB19630258, could not be aligned with the sequences of the five new species, showing that these new species do not belong to section *Myxotelamonia*. The most closely related sequences from Moser and Horak´s South American holotypes were *C. mitis* (Subgenus *Myxacium,* Section *Ochroleuci*), which is related to the clade with the new species *C. neuquensis* (BPP = 0.83); *C. micaceus* (subgenus *Sericeocybe* strips *Nothoanomalus)*, *C. cinereus* (Section *Telamonia*) and *C. nitellinus* (Section *Formiores*) are weakly related to the new species *C. voluptatis* (BPP = 0.77); and *C. avellaneus* (Section *Myxotelamonia*)*, C. semiamictus* (Subgenus *Paramyxacium,* stirps *Myxacioides)* and *C. macilentus* (Section *Myxotelamonia*) are related to the new species *C. gracilentus* (BPP = 1.00). 

The concatenated analysis of the LSU-ITS rDNA and RPB1 regions (best ML tree with log likelihood −17366.88) indicates a possible common origin for *C. pseudoxiphidipus, C. voluptatis* and *C. egonii* (BPP 0.929). Our data also suggest that *C. neuquensis* could be related to a clade containing *C. lustratus* Fr.*, C. cretax* Soop and *C. pinophilus* Soop (BPP 0.871) (Supplementary Figure S1). 

### 3.2. Taxonomic Data

**Figure 2:**
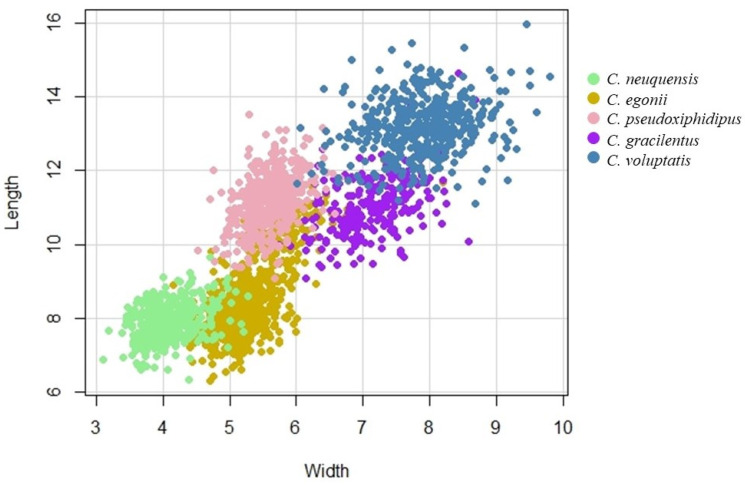
Scatterplot of spore length and width for the five newly described *Cortinarius* species.

**Figure 3:**
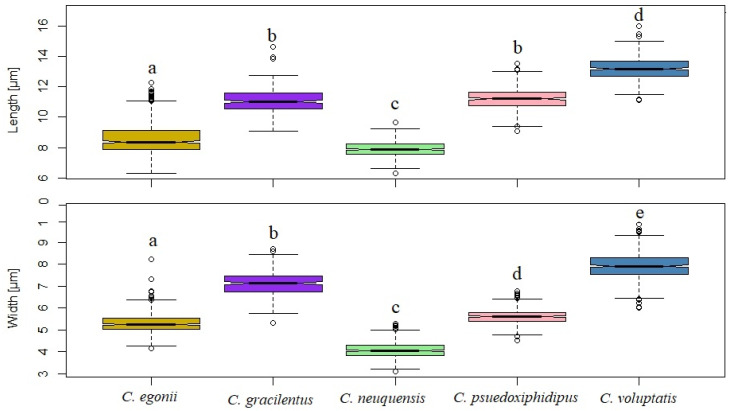
Boxplot of spore lengths and widths for the five newly described *Cortinarius* species. Different letters indicate significant differences in the means (*p* < 0.0001) based on Kruskall Wallis tests.

All the species included in the morphological study differ significantly in the dimensions of their spores (Kruskal Wallis, H = 1957.22, *p* < 0.0001), confirming statistically different clouds of data (Figure 2). Statistical analysis of basidiospore measurements confirmed that the spores of *C. neuquensis* are significantly smaller (in both width and length) than spores of other *Cortinarius* species from this study. On the other hand, *C. voluptatis*’ spores are significantly bigger (in both width and length) from any other species in this study (Kruskal Wallis, H = 1957.22, *p* < 0.0001, Figure 3). All species can clearly be separated from each other based on their spore size and shape (as a function of Q = length/with).

### 3.3. Taxonomy

#### 3.3.1. *Cortinarius egonii* Salgado Salomón, Peintner, Liimat. and *Niskanen* spp. nov.

#### MycoBank MB 836828

#### Etymology

The species epithet refers to Dr. Egon Horak, globally recognized expert on the genus *Cortinarius*. His work has inspired many mycologists around the world to discover the fascinating world of *Cortinarius* taxonomy, including the authors of this paper.

#### Diagnosis 

**Figure 4:**
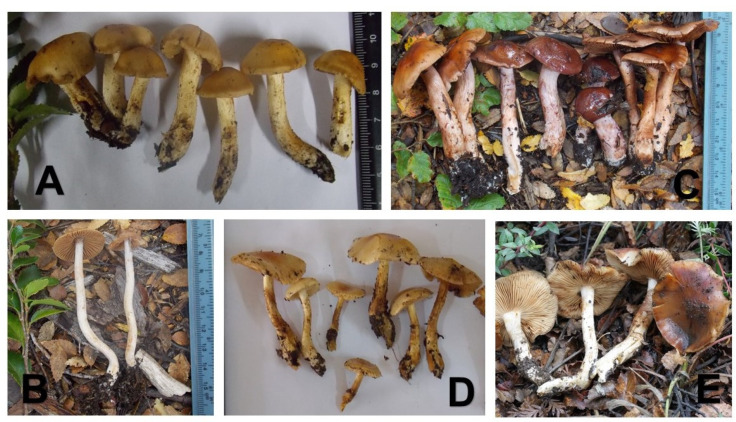
New *Cortinarius* species proposed. Photos of basidiomata. (**A**) *C. pseudoxiphidipus*. (**B**) *C. gracilentus*. (**C**) *C. voluptatis*. (**D**) *C. egonii*. (**E**) *C. neuquensis*. Photos (**A**–**D**) by MESS, photo E by PB Matheny.

**Figure 5:**
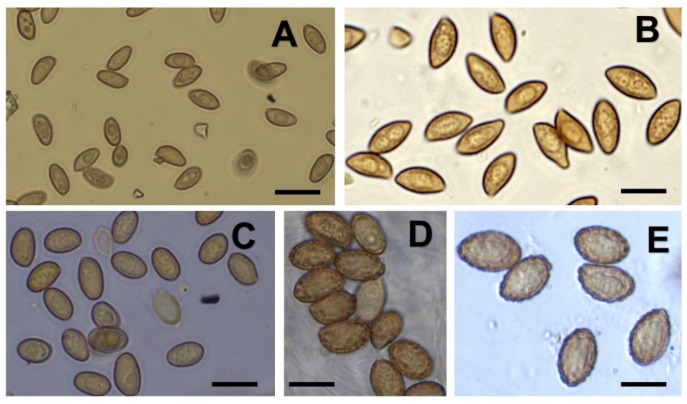
Basidiospores of the new *Cortinarius* species proposed. Photos of (**A**) *C. neuquensis*. (**B**) *C. pseudoxiphidipus*. (**C**) *C. egonii*. (**D**) *C. gracilentus*. (**E**) *C. voluptatis*. The bar represents 10 µm.

*Cortinarius egonii* (Figure 4D and Figure 5C) has medium-sized basidiomata (pileus: 2.7–4.4 cm in diam.; stipe: 3.7–5.0 × 0.5–0.6 cm) and is characterized by a glutinous pileus with pale yellow to maize colors and a darker, brown center; elliptical, inconspicuously verrucose (6.3)7.5–8.9(10.4) × (4.2)4.8–5.5(6.6) µm spores and the presence of melanized, thick-walled hyphae with a diam. of (3)4–7 µm in the context.

#### Type

Argentina, Río Negro, Bariloche, Nahuel Huapi National Park, Steffen lake, Coordinates Lat.: −41.3062; Long.: −71.3207; Alt.: 571.5 m.a.s.l. Associated with Nothofagus dombeyi, leg. et det. María Eugenia Salgado Salomón, Holotype: HCFC C257; Isotype: IB 20170257, 16 May 2017, Genbank acc. No. MN707589.

#### Macrocharacters

PILEUS 2.7–4.4 cm in diam., convex in young specimens, hemispherical to convex and plano-convex with age, pileus margin slightly bent, in young specimens slightly involute. Pileus surface glutinous, slightly hygrophanous, smooth. Pileus color varies between cream (9D2) to cork (13B7) at the margin with clearly darker colors towards the pileus center ranging between artificial brown (8L6) and Tuscany brown (7L11–7L12), later mixed with an orange tone.

LAMELLAE sinuated to adnate, on average dense, about 14–15 lamellae per cm, margin entire to finely eroded. Color of the lamellae at the pileus margin when young cream (9D2) to corn (10J5), becoming terracotta colored with age (4D12).

STIPE 3.7–5.0 × 0.5–0.6 cm, cylindrical to clavate, longitudinally fibrous, dry, white (10B1–10C1), with yellowish with remnants of a universal veil; Cortina evanescent, white in young specimens, disappearing with age.

Context ochraceous when fresh, with paler colors towards the margin of the stipe. Smell fungal to sweetish in gills. Taste mild. Usually growing in groups but not cespitose.

Macrochemical reactions: 20% KOH negative on exsiccate. No fluorescence was detected at 350 nm nor at 254 nm (in exsiccate material).

#### Microcharacters

Basidiospores (6.3) 7.5–8.9 (10.4) × (4.2) 4.8–5.5 (6.6) µm. (mean ± sd: 8.2 ± 0.5 × 5.2 ± 0.3 µm, Q: (1.3) 1.6 ± 0.2 (2.1); (*n* = 142) for holotype. Elliptical, subcylindrical, very slightly ornamented, inconspicuously verrucose, not dextrinoid, pale bronze brown.

BASIDIA with four sterigmata, occasionally 2-sterigmata and basal clamp connection, clavate, (26) 27–30 (31) × (6) 7–9 µm, sterigmata 3–4 µm long (*n* = 10).

CHEILOCYSTIDIA present, clavated, (31) 32–37 (39) × 6–9 µm (*n* = 16).

LAMELLAR TRAMA consisting of parallel hyaline clamped hyphae with a diam. of (4)5–8(11) µm (*n* = 25), thin walled.

PILEIPELLIS with a 160 µm wide gelatinous layer, consisting of hyaline hyphae with a diam. of 4–6 µm (*n* = 20) and clamp connections.

PILEUS CONTEXT 800–900 µm wide, formed by two layers. Uppermost with inflated hyphae elements with a diam. of (11)15–31 (45) µm (*n* = 37), spongy looking, hyaline, colorless, thin-walled, hyphal walls not encrusted. Intermediate layer with hyphae of a diam. of (8)9–12(15) µm (*n* = 30), hyaline, colorless, thin-walled, hyphal walls not encrusted. None of the two layers is staining with 3% KOH, cotton blue, Melzer’s reagent or Sulpho-vanillin. Within the regular layer of the context, there are non-clamped oleiferous-like hyphae of amber colors (10I6 to 10J6; in 3% KOH) they are thick-walled and somewhat irregular with a diam. of (3)4–6(7) µm (*n* = 16). They do not stain with cotton blue, Melzer’s reagent or Sulpho-vanillin.

Clamp connections present in all tissues.

#### Ecology and Distribution

Forest type—*Nothofagus dombeyi, N. pumilio* and *N. antarctica*; observed in May. The monthly average temperature for May in the area is 8 °C (max/min 13/5 °C), with a total of 95 mm precipitation (weather station El Bolsón Aero, data from 2017). Soil pH = 5.8.

*Other material examined:* Argentina, Chubut, Futaleufú, Los Alerces National Park, Arroyo Colihual, Coordinates Lat.: −42.7005 W; Long.: −71.7041. Associated with *Nothofagus dombeyi*, leg. et det. María Eugenia Salgado Salomón, herbaria number HCFC C52/IB 20170324; 11 April 2017, Genbank acc. No. MN707571.

Argentina, Río Negro, Bariloche, Nahuel Huapi National Park, Steffen lake, Coordinates Lat.: −41.3071; Long.: −71.3237; Alt.: 551 m.a.s.l. Associated with *Nothofagus dombeyi*, leg. et det. María Eugenia Salgado Salomón, herbaria number HCFC C246/IB 20170447; 16 May 2017, Genbank acc. No. MN707588.

Argentina, Río Negro, Bariloche, Nahuel Huapi National Park, Steffen lake, Coordinates Lat.: −41,3062; Long.: −71,3207; Alt.: 571.5 m.a.s.l. Associated with *Nothofagus dombeyi*, leg. et det. María Eugenia Salgado Salomón, herbaria number HCFC C258/IB 20170258; 16.05.2017, Genbank acc. No. MN707590.

Argentina, Río Negro, San Carlos de Bariloche, Nahuel Huapi National Park, Arroyo Goye, near Colonia Suiza, Coordinates Lat.: −42.7005 W; Long.: −71.7041. Associated with *Nothofagus dombeyi* and *N. pumilio*, leg. Tuula Niskanen et al., herbaria number MES-1888, CORDC00005629; 12.05.2016, Genbank acc. No. KY462608.

Argentina, Río Negro, Bariloche, Nahuel Huapi National Park, Road to Tronador, just before the mountain base. Associated with *Nothofagus pumilio*, leg. Tuula Niskanen et al., herbaria number MES-2001, CORDC00005551; 14 May 2016, Genbank acc. No. MT925622.

Argentina, Río Negro, Bariloche, Nahuel Huapi National Park, Los Rápidos. Associated with *Nothofagus dombeyi* and *N. antarctica*, leg. P. Brandon Matheny, herbaria number MES-1205; 11 May 2016, Genbank acc. No. MT925623.

Argentina, Río Negro, Bariloche, Nahuel Huapi National Park, Los Rápidos. Associated with *Nothofagus antarctica*, leg. Tuula Niskanen et al., herbaria number MES-1930, CORDC00005614; 13 May 2016.

Chile, Aysen, Carretera Austral, south of Bertrand port. Coordinates lat.: −47.0629; long.: −72.8008. Associated with managed *Nothofagus pumilio* and *N. dombeyi* forests, leg. C. Truong, herbaria number CT-4418, FLAS-F-63487; 03 May 2016, Genbank acc. No. MT925625.

Notes: *Cortinarius egonii* has a mean of 0 bp within species variation in the ITS region, except for collection HCFC C80, that differs from the ITS sequence of the holotype by 0.7% (five substitutions and indels). *C. egonii* is the only representative of the UNITE SH1142013.08FU and differs by 4% (22 substitutions and indels) from the most closely related reference sequence *C. rhodophyllus* (GenBank Acc. No. KJ421051, Chile). The difference to all other species ranges between 4.5 and 9.0% (29 and 50 substitutions and indels) (MW = 33, SD = 6).

*Cortinarius rhodophyllus* was placed by Moser and Horak (1970) in subgenus *Phlegmacium,* section *Calochroi*. *C. rhodophyllus* clearly differs from *C. egonii* by having a bulbous stipe, reddish–salmon lamellae and amygdaliform spores. Morphologically, *C. luteocaeruleus* Moser somewhat resembles *C. egonii*. However, this species clearly differs from *C. egonii* by the bluish colors at the stipe apex, the red KOH reaction on the pileus, and distinctly larger basidiospores (9.5–11 × 6–6.7 µm). A sequence is not available for *C. luteocaeruleus*. *Cortinarius punctatisporus* Garnica morphologically also resembles *C. egonii* but differs with a clearly inflated stipe. Moreover, the ITS sequence similarity of *C. egonii* and *C. punctatisporus* is < 97% [8,38].

#### 3.3.2. *Cortinarius gracilentus* Salgado Salomón and *Peintner* spp. nov.

#### MycoBank MB 836579

#### Etymology

The species epithet refers to the slender habitus of the basidiomata.

#### Diagnosis

*Cortinarius gracilentus* (Figrue 4B and Figrue 5D) is characterized by a glutinous, hygrophanous, cinnamon brown pileus, stipe cylindrical, dry, fibrous, white, with the remains of a caramel veil, and cocoa brown lamellae in young specimens. Basidiospores are elliptic, verrucose, 11.1 ± 0.7 × 7.1 ± 0.5 µm. Basidia stain with cotton blue and usually grow alone.

#### Type

Argentina, Chubut, Futaleufú, Los Alerces National Park, Río Rivadavia, Coordinates Lat.: −42.4002; Long.: −71.4081; Alt.: 502.1. Associated with *Nothofagus dombeyi*, leg. et det. María Eugenia Salgado Salomón, Holotype HCFC C66; Isotype: IB20170234, 18 April 2017, Genbank acc. No. MN707572.

#### Macrocharacters

PILEUS (1.9) 2.3–3.3 cm diam., convex to campanulate, becoming low convex with age. The margin of the pileus is plane. Pileus surface glutinous, hygrophanous, smooth. Pileus color varies between cinnamon (12A5 to 12E7) at the margins and amber brown (12L12 to 13K12) at discal zone.

LAMELLAE sinuate, subdistant, about 6–9 lamellae per cm at the pileus margin, margin finely eroded, color like milky tea (11B7 to 11C6), becoming amber brown (13K12).

STIPE (4.5) 4.8–7.8 (8.4) × 0.5 cm, cylindrical to weakly clavate, dry, cartilaginous, whitish (9A1), with the remains of a caramel-colored universal veil (12F10) especially in the middle part; Cortina white silky in young specimens, evanescent. Brown rhizomorphs present.

CONTEXT (flesh) firm, corky with pale colors. Smells inconspicuous, slightly of fungi. Usually grow as single fruiting bodies. Macrochemical reactions; 20% KOH on exsiccate slightly yellowish. No fluorescence was detected at 350 nm nor at 254 nm (in dry material).

#### Microcharacters

Basidiospores (9.1) 10.4–11.8 (13.8) × (5.3) 6.6–7.6 (8.7) µm (mean ± sd: 11.1 ± 0.7 × 7.1 ± 0.5 µm), Q: (1.2) 1.6 ± 0.1 (2); (*n* = 131) for the holotype elliptical, verrucose, not dextrinoid, cocoa brown to coffee brown.

BASIDIA with four sterigmata and a basal clamp, clavate, (37)39–44(47) × (10)11–12(13) µm (*n* = 28), sterigmata 5–7(8) µm long (*n* = 49), cyanophilous with cotton blue.

CYSTIDIA not observed.

LAMELLAR TRAMA consisting of parallel hyaline thin-walled hyphae with clamp connections and a diam. of 5–7 (8) µm (*n* = 35).

PILEIPELLIS with a 200 µm thick gelatinous layer of pileus context consisting of clamped hyaline hyphae with a diam. of 4–6(8) µm (*n* = 35), hyphae stain with 3% KOH with yellowish colors (10I6 to 10J6).

PILEUS CONTEXT 800–900 µm wide, formed by two layers, the uppermost with inflated hyphae elements with a diam. Of (11) 15–31 (45) µm (*n* = 37), spongy looking, hyaline, colorless, thin-walled, hyphal walls not encrusted. The intermediate layer has hyphae of a diam. of (8) 9–12 (15) µm (*n* = 30), hyaline, colorless, thin-walled, hyphal walls not encrusted. None of the two layers are staining with 3% KOH, cotton blue, Melzer’s reagent or Sulpho-vanillin. Within the regular layer of the context, there are non-clamped oleiferous-like hyphae of amber colors (10I6 to 10J6; in 3% KOH) they are thick-walled and somewhat irregular with a diam. of (3) 4–6 (7) µm (*n* = 16). They do not stain with cotton blue, Melzer’s reagent or Sulpho-vanillin.

Clamp connections present in all tissues.

#### Ecology and Distribution

Forest type—*Nothofagus dombeyi* and *N. antarctica;* observed in April. The monthly average temperature in the area is 8.9 °C (max/min 13.7/2 °C), with a total of 82 mm precipitation in April (weather station Lago Cholila, data from 2017). Soil pH = 5.8.

*Other material examined:* Argentina, Chubut, Futaleufú, Los Alerces National Park, Camping trail, coordinates Lat.: −42.4001; Long.: −71.4087; Alt.: 521.5 m.a.s.l., associated with *Nothofagus antarctica*, leg. et det. María Eugenia Salgado Salomón. Herbaria number HCFCC171, IB 20170235, 18 April 2017. Genbank acc. No. MN707580.

Notes: *Cortinarius gracilentus* has no within species ITS differences and differs by 0.1% (one substitution or indel) from the included subclade (MES-1597, MES-1801). The most closely related species is *C. avellaneus* Moser, which differs by 4.5% in the ITS region (23 substitutions or indels). The difference to all other species ranges from 4.5 to 9% (23–49 substitutions or indels MW 31 + −8 bp).

Based on ITS sequences only, where the majority of reference sequences are available, *C. gracilentus* is closely related to *C. avellaneus.* Morphological characters confirm this relationship: *C. avellaneus* has darker avellaneous to umber brow dry pilei, yellow-rusty brown lamellae and further differs by ellipsoid to amygdaliform, strongly verrucose spores with an inconspicuous plage. The second species belonging to this stirps Avellaneus, *C. fulvoconicus* Moser, differs by the vividly red-brown pileus colours, the dry pileus, and narrower spores. Moser and Horak (1977) stated that stirps *Avellaneus* is closely related to stirps *Rufus* as confirmed by the sister group relationship to *C. rufus* Moser and *C. subrufus* San-Fabian, Niskanen & Liimat. The stirps *Rufus* has been validated as sect. *Austroamericani* (San Fabian et al. 2018).

#### 3.3.3. *Cortinarius neuquensis* Salgado Salomón, Peintner, Liimat. and Niskanen spp. nov.

#### MycoBank MB 836823

#### Etymology

Epithet refers to Neuquén province (Argentina), where the first vouchers used for this species description were collected.

#### Diagnosis

*Cortinarius neuquensis* (Figrue 4E and Figrue 5A) has medium-sized basidiomata (3.5–5 cm pileus diam.) and is characterized by a light ochre brown to reddish brown, glutinous, not hygrophanous pileus, and a white, cylindrical, fragile, and dry stipe. The lamellae are pale argillaceous, with an entire margin and sinuate. Basidiospores are amygdaliform to elliptical and finely verrucose, (6.3) 7.3–8.3 (9.7) × (3.1) 3.6–4.1 (5.3) µm. Basidia stain with cotton blue. In the external layer of context there are thick-walled hyphae that stain in cotton blue and do not have clamp connections.

#### Type

Argentina, Neuquén, Aluminé, Chañy Natural Protected Area, Arroyo Chañy, Coordinates Lat.: −38.9456; Long.: −71.3139; Alt.: 1165.5, in mixed forests of *Nothofagus antarctica* and *Araucaria araucana,* leg. et det. María Eugenia Salgado Salomón, Holotype: HCFC C206; Isotype: IB 20170222, 04 May 2017, Genbank acc. No. MN707582.

#### Macrocharacters

PILEUS 3.6–5.0 cm in diam., low convex, occasionally conical in young specimens, pileus margin somewhat wavy with age. Pileus surface glutinous, not hygrophanous, smooth. Pileus color varies between light ochre (10C1), golden light brown (10H4) to reddish brown, becoming with age honey ochre (12J6). The margin of the pileus is straight, and in young specimens slightly bent.

LAMELLAE sinuate, on average dense, about 12–14 lamellae per cm at the pileus margin, margin entire, pale argillaceus (9E4) to argillaceus (9E7), later cinnamon brass colored (11L6-11L9).

STIPE 3.5–7.5 × 0.7–1.1 cm, cylindrical to somewhat rooting, corky, fragile, dry, white (9G1–9H1), with remnants of a pale cinnamon universal veil, especially in the middle part. Cortina white, quickly evanescent, when present, sparse, brown–ferruginous from the spore deposit (12L9).

Smell in lamellae, strong, spicy, sometimes slightly ammoniacal. Tastes mild. Usually grows alone.

Macrochermical reactions; 20% KOH, negative on dry basidiomes. No fluorescence was detected at 350 nm nor at 254 nm (in exsiccate material).

#### Microcharacters

Basidiospores (6.3) 7.3–8.3 (9.7) × (3.1) 3.6–4.1 (5.3) µm (mean ± sd: 7.8 ± 0.5 × 3.9 ± 0.3 µm, Q: (1.4) 2 ± 0.2 (2.4); (*n* = 125) for holotype (measured from the stipe or cortina), pale golden brown, amygdaliform to elliptical, finely verrucose, not dextrinoid.

BASIDIA with four sterigmata and a basal clamp, clavate, (23) 24–30 (33) × 6–7 µm, sterigmata 2–4 (5) µm long (*n* = 16), cyanophilous with cotton blue. Does not stain with KOH nor Sulpho vanillin.

CYSTIDIA not observed.

LAMELLAR TRAMA consisting of parallel thin-walled hyaline hyphae with a diam. of (6) 7–11 (15) µm (*n* = 31).

PILEIPELLIS a cutis of regular, slightly interwoven hyphae, approximately 170 µm thick, uppermost layer gelatinous, hyphae hyaline, with a diam. of 2–3 (5) µm (*n* = 20).

PILEUS CONTEXT consisting of two layers. External layer with regular hyphae elements with a diam. of (8) 11–19 (25) µm (*n* = 22) hyaline, colorless, thin-walled, hyphal walls not encrusted, not staining with 3% KOH, cotton blue, Melzer’s reagent or Sulpho-vanillin, approximately 450 µm thick. Internal layer with globose hyphae elements, diam. of (22) 25–36 (46) µm (*n* = 20) hyaline, colorless, thin-walled, hyphal walls not encrusted, not staining with 3% KOH, cotton blue, Melzer’s reagent or Sulpho-vanillin, approximately 250 µm thick. In the external layer oleiferous-like unclamped thick-walled hyphae with a diam. of (3) 4–5 µm (*n* = 10) are present. These hyphae run longitudinal to context, and are cyanophilous, but do not stain with Melzer’s reagent or Sulpho-vanillin.

Clamp connections present in all tissues.

#### Ecology and Distribution

Forest type—mostly in forest of *Nothofagus antarctica*, but also in forests of *N. dombeyi* and in mixed forests of *Lophozonia alpina-L. obliqua*; observed during May. The monthly average in May temperature in the area is 9 °C (max/min 14/6 °C), with a total of 88 mm precipitation (weather station Lago Ñorquinco, data from 2017). Soil pH = 6.1. This species has also been detected in Chile (KY462703, KY462509)

*Other materialexamined*: Argentina, Neuquén, Aluminé, Lanín National Park, Ñorquinco Lake, coordinates Lat.: −39.0877; Long.: −71.1546; Alt.: 1060.1 m.a.s.l., associated with mixed forest of *Lophozonia alpina* and *L. obliqua,* leg. et det. María Eugenia Salgado Salomón. Herbaria number HCFCC196, IB 20170218, 3 May 2017. Genbank acc. No. MN707581.

Argentina, Neuquén, Aluminé, Chañy Natural Protected Area, Chañy stream, Coordinates Lat.: −38.9456; Long.: −71.3139; Alt.: 1165.5. associated with mixed forests of *Nothofagus antarctica* and *Araucaria araucana,* leg. et det. María Eugenia Salgado Salomón, herbaria number HCFC C210, IB 20170222, 4 May 2017, Genbank acc. No. MN707583.

Chile, Osorno, Puyehue National Park, foothills of Volcan Puyehue, up the road past el Caulle north of Río Golgol, associated with *Nothofagus dombeyi,* leg. Tuula Niskanen et al., herbaria number MES-1638, 234,303 (k), FLAS-F-64429; 4 May 2016, Genbank acc. No. KY462509.

Argentina, Río Negro, Bariloche, Nahuel Huapi National Park, road to tronador, at the end of lake Muscardi before Pampa Linda. Associated with *Nothofagus antarctica,* leg. Tuula Niskanen et al., herbaria number MES-2009, CORDC00005547; 14 May 2016, Genbank acc. No. MT925951.

Argentina, Río Negro Bariloche, Nahuel Huapi National Park, along road halfway to Tronador. Associated with *Nothofagus antarctica,* leg. Brandon Matheny, herbaria number MES-1148, CORDC00005190; 9 May 2015, Genbank acc. No. MT925952.

Chile, Osorno, Puyehue National Park, near Aguas Calientes inside the national park. Associated with *Nothofagus dombeyi,* leg. Tuula Niskanen et al., herbaria number MES-1551, K(M)234248, FLAS-F-64363; 3 May 2016, Genbank acc. No. MT925953.

Notes: *Cortinarius neuquensis* has a mean within species ITS difference of 0–0.7% (0–4 substitutions and indels) and differs by 2–3% (12–17 substitutions and indels) from *C. verniciorum* Soop, and by 2.5% (14–15 substitutions and indels) from *C. perelegans* Soop. The difference from all other species ranges 5–8% (29-50 substitutions and indels, MW = 32, SD = 6). *Cortinarius verniciorum* and *C. ducamarus* Soop from New Zealand belong to the same section *Verniciori* Soop. These two species differ by orange brown basidiomes with strongly viscid pilei. *Cortinarius viscovenetus* Horak is morphologically similar. However, the spores are significantly larger (10–11 × 6.8–7.2 µm). The ITS sequence similarity of these two species is <97% (unpublished). 

#### 3.3.4. *Cortinarius pseudoxiphidipus* Salgado Salomón and *Peintner* spp. nov.

#### MycoBank MB 836577

#### Etymology

The epithet refers to its morphological affinity to *Cortinarius xiphidipus.*

#### Diagnosis

*Cortinariuspseudoxiphidipus* (Figrue 4A and Figrue 5B) is characterized by glutinous, hygrophanous, honey brown pileus, subclavately radicant, dry, fibrous, whitish stipe with pale copper veil remaining and concolorous, copper-colored lamellae in young specimens. Basidiospores are elliptical, finely verrucose, 11.4 ± 0.8 × 5.6 ± 0.3 µm, hymenial cystidia not present. Usually growing in scatted groups.

#### Type

Argentina, Chubut, Futaleufú, Los Alerces National Park, Río Rivadavia, Coordinates Lat.: −42.4003; Long.: −71.4107; Alt.: 511.7. Associated with *Nothofagus dombeyi*, leg. et det. María Eugenia Salgado Salomón, Holotype (CIEFAP) HCFCC88; Isotype (IB): IB 20170347, 19 April 2017, Genbank acc. No. MN707575.

#### Macrocharacters

PILEUS (2.2) 2.4–4.4 (5) cm diam., convex, becoming low convex with age. The margin of the pileus is slightly bent, in young specimens slightly involute, becoming alveolate, with deep indentations. Pileus surface glutinous, hygrophanous, smooth. Pileus color varies between honey brown (11K6 to 11K5, Rattan) to amber brown (13K12 to 13J12) in discal zone.

LAMELLAE sinuate, on average dense, about 12–14 lamellae per cm. at the pileus margin, margin finely eroded, with copper colors (11L6, brass), later with wild honey colors (13L9).

STIPE (3.1) 4–5.8 (6.6) × 0.7–1.3 (1.8) cm, radicant to weakly cylindrical, bent, dry, fibrous, whitish (10A1), with remnants of pale copper veil, especially in the middle part. Cortina white silky in young specimens, evanescent.

CONTEXT (flesh) firm, corky with pale colors. Smells slightly fruity. Tastes mild.

Usually growing in scattered small groups.

Macrochemical reactions; 20% KOH on exsiccate material slightly yellowish. Yellowish reaction on exsiccate lamella with 3% KOH. No fluorescence was detected at 350 nm nor at 254 nm (in exsiccate material).

#### Microcharacters

Basidiospores (9.1) 10.6–12.2 (13.5) × (4.5) 5.3–6 (6.7) µm (mean ± sd: 11.4 ± 0.8 × 5.6 ± 0.3 µm), Q: (1.6) 2 ± 0.1 (2.6); (*n* = 159) amygdaliform to sublimoniform, finely verrucose, not dextrinoid, golden brown to cocoa brown (for holotype, measured from the stipe or cortina). Basidiospores on lamellae heterogeneous in size when immature usually smaller and paler than mature basidiospores.

BASIDIA with four sterigmata and a basal clamp, clavate, (34) 36–40 (41) × 9–11 (12) µm, sterigmata 4–6 µm long (*n* = 10). Some basidia with oily yellow content when observed in KOH (3%). Does not stain with cotton Blue, Melzer’s reagent or Sulpho vanillin.

CYSTIDIA not observed.

LAMELLAR TRAMA consisting of parallel hyaline thin-walled hyphae with clamp connections and a diam. of (2) 4–5(6) µm (*n* = 30).

PILEIPELLIS with a gelatinous layer, pileus context consisting of clamped hyaline hyphae with a diam. of 4–6 (7) µm (*n* = 14).

PILEUS CONTEXT 350 to 400 µm wide, formed by two layers, The uppermost with regular hyphae elements with a diam. Of (12) 13–19 (23) µm (*n* = 15) hyaline, colorless, thin-walled, hyphal walls not encrusted. The intermediate layer has inflated hyphae elements with a diam. of (32) 34–39 (40) µm (*n* = 10) hyaline, colorless, thin-walled, hyphal walls not encrusted. None of layers are staining with 3% KOH, cotton blue, Melzer’s reagent or Sulpho vanillin. Clamp connections present in all tissues.

#### Ecology and Distribution

Forest type—*Nothofagus dombeyi*; observed in April. The monthly average temperature in the area is 8.9 °C (max/min 13.7/2 °C), with a total of 82 mm precipitation in April (weather station Lago Cholila, data from 2017). Soil pH = 5.8.

*Other materialexamined*: Argentina, Chubut, Futaleufú, Los Alerces National Park, Río Rivadavia trail, coordinates Lat.: −42.4002; Long.: −71.4108; Alt.: 502.1 m a.s.l., associated with *Nothofagus dombeyi,* leg. et det. María Eugenia Salgado Salomón. Herbaria number HCFCC78, IB 20170340, 18.04.2017. Genbank acc. No. MN707573.

Argentina, Chubut, Futaleufú, Los Alerces National Park, Río Rivadavia trail, coordinates Lat.: −42.4003; Long.: −71.4107; Alt.: 511.7 m.a.s.l., associated with *Nothofagus dombeyi,* leg. et det. María Eugenia Salgado Salomón. Herbaria number HCFCC90, IB 20170441, 18.04.2017. Genbank acc. No. MN707576.

Notes: *Cortinarius pseudoxiphidipus* has a mean within species ITS difference of 0 bp and differs by >5% (28–48 substitution and indels MW 29 ± 4 bp) from the other species included in the phylogeny, and the difference to *C. xiphidipus,* which it resembles morphologically, is >6%. *Cortinarius pseudoxiphidipus* has smaller basidiomes, lamellae with copper colors, and larger basidiospores than *C. xiphidipus. Cortinarius xiphidipus* has argillaceous lamellae, and elliptical, finely warty spores of 6–8 × 4–4.8 µm.

#### 3.3.5. *Cortinarius voluptatis* Salgado Salomón and *Peintner* spp. nov.

#### MycoBank MB 836578

#### Etymology

From Latin, the one who provides pleasure or joy. The species epithet refers to the red wine colors of the pileus in young specimens.

#### Diagnosis

*Cortinarius voluptatis* (Figrue 4C and Figrue 5E) is characterized by glutinous, red wine pileus, subclavate, dry, fibrous, whitish stipe with remaining of red-violaceus veil and lilaceous lamellae in young specimens. Basidiospores elliptic, verrucose, 13.1 ± 0.7 × 8.0 ± 0.5 µm, subclavate cheilocystidia present. Usually growing in small groups.

#### Type

Argentina, Neuquén, Lácar, San Martín de los Andes, Lanín National Park, Yuco, Coordinates Lat.: −40.09974; Long.: −71.31527; Alt.: 657.5. Associated with *Lophozonia alpina* and *L. obliqua*, leg. et det. María Eugenia Salgado Salomón, Holotype (CIEFAP) HCFCC219; Isotype (IB): IB 20170231, 5 May 2017, Genbank acc. No. MN707585.

#### Macrocharacters

PILEUS (4.3) 4.8–6.4 (6.7) cm diam., convex when young, becoming applanate with age. The margin of the pileus is slightly bent, in young specimens slightly involute, becoming undulate. Pileus surface glutinous, hygrophanous, smooth. Pileus color varies between red wine (7J6 to 7L10, Spanish wine), later with Hazel colors (12I7 to 13J9, Hazel) and more vivid, gold–brown color in the discal zone (14F12).

LAMELLAE sinuate, on average dense, about 12–14 lamellae per cm at the pileus margin, margin finely eroded, light violaceous (54A5 to 55A5, La Valière), becoming light brown (13A6).

STIPE (6.2) 6.8–8.2 (8.5) × 0.7–1.3 (1.4) cm, subclavate to cylindrical, dry, fibrous, whitish (10A1), with the remains of a red–violaceus veil, especially in the middle part which gives the whole stipe a lilac hue when young, white cortina, silky in young specimens, evanescent.

PILEUS CONTEXT (flesh) firm, cartilaginous with pale colors. Smells sweetish ammoniacal. Taste not detected.

Usually growing in scattered small groups.

Macrochemical reactions; 20% KOH on exsiccate. Pileus slightly yellowish. No fluorescence was detected at 350 nm nor at 254 nm (in exsiccate material).

#### Microcharacters

Basidiospores (11.6) 12.4–13.7 (15.9) × (6.5) 7.5–8.5 (9.8) µm (mean ± sd: 13.1 ± 0.7 × 8 ± 0.5 µm), Q: (1.3) 1.6 ± 0.1 (2); (*n* = 141) for holotype (measured from the stipe or cortina) Elliptical to subamygdaliform, clearly verrucose, without plage, not dextrinoid, coffee to cocoa brown. Basidiospores on lamellae heterogeneous in size, when immature usually smaller and paler than mature basidiospores.

BASIDIA with four sterigmata and basal clamp, clavate, (33) 34–38 × 9–12 (13) µm, sterigmata 3–5 µm long (*n* = 10).

CHEILOCYSTIDIA present, slightly clavate, 34–39 (40) × 10–12 µm (*n* = 10), scarce.

LAMELLAR TRAMA consisting of parallel hyaline thin-walled hyphae with clamp connections and a diam. Of (4) 6–10 (12) µm (*n* = 32).

PILEIPELLIS with a 270 µm wide gelatinous layer of consisting of clamped hyaline hyphae with a diam. Of (4) 5–6 (7) µm (*n* = 40).

CONTEXT 650 µm approx. wide, with inflated hyphae elements, hyaline, colorless, thin-walled, hyphal walls not encrusted with a diam. Of (17)18–40 µm (*n* = 10), the first 130 µm of context staining yellowish with 3% KOH. This layer amber in color (10I6 to 10J6; in 3% KOH); thick-walled, irregular, no clamped oleiferous-like hyphae with a diam. Of 5–8 (10) µm (*n* = 11). No elements of the context are staining with cotton blue, Melzer’s reagent or Sulpho vanillin.

Clamp connections present in all tissues.

#### Ecology and Distribution

Forest type—*Lophozonia alpina* and *L. obliqua* but occasionally with *N. antarctica*; observed in May. The monthly average temperature in the area is 7 °C (max/min 12/2 °C), with a total of 136 mm precipitation in May (weather station Chapelco Aero, data from 2017). Soil pH = 5.8.

*Other material examined*: Argentina, Chubut, Futaleufú, Los Alerces National Park, Rivadavia Camping area, coordinates Lat.: −42.4006, Long.: −71.4073; Alt.: 506.8 m.a.s.l. Associated with *Nothofagus antarctica*, leg. et det. María Eugenia Salgado Salomón, CIEFAP herbarium HCFCC157; IB 20170109, 25 April 2017, Genbank acc. No. MN707579.

Argentina, Neuquén, Lácar, San Martín de los Andes, Lanín National Park, Yuco, coordinates Lat.: −40.0997, Long.: −71.3154, Alt.: 657.5 m.a.s.l. Associated with *Lophozonia alpina* and *L. obliqua*, leg. et det. María Eugenia Salgado Salomón, CIEFAP herbarium HCFCC218; IB 20170229, 5 May 2017, Genbank acc. No. MN707584.

Argentina, Neuquén, Lácar, San Martín de los Andes, Lanín National Park, Yuco, coordinates Lat.: −40.0989, Long.: −71.3147, Alt.: 653.6 m.a.s.l. Associated with *Lophozonia alpina* and *L. obliqua*, leg. et det. María Eugenia Salgado Salomón, CIEFAP herbarium HCFCC230; IB 20170238, 5 May 2017, Genbank acc. No. MN707587.

Notes: *Cortinarius voluptatis* has a mean within species ITS difference of 0.1% (one substitution or indel) and differs by 2.3% (13 substitutions or indels) from the most closely related species *C. cinereus* Moser. The difference to all other species ranges between 3 and 9% (17 and 49 substitutions or indels MW = 27, SD = 7).

Based on morphology, *C. voluptatis* could belong to Section *Myxotelamonia* Subsect. *Lilacifolii* [5]. The type of the section, *C. roseopurpurascens* Moser & Horak, somewhat resembles *C. voluptatis,* but differs by the stipe being lilaceous, it has significantly smaller spores (10–12.5 × 6.5–7.5 µm) and it lacks a veil. Moser & Horak [5] hypothesized that this section could represent an endemic South American complex of species without morphologically similar species in the Northern Hemisphere. Old specimens of *Cortinarius voluptatis* could be confused with *C. pseudoxiphidipus* because of the size and fruiting habit, but *C. voluptatis* can be differentiated by the darker color of the veil and the larger spores. *Cortinarius juglandaceus* Soop resembles *C. voluptatis* but is not closely related. *C. juglandaceus* is viscid whereas *C. voluptatis* has a glutinous pileus surface and has larger spores [54]. *Cortinarius juglandaceus* occurs in *Nothofagus* forests in New Zealand. 

## 4. Discussion

Five new species of *Cortinarius* are proposed here based on morphological and molecular data. *Cortinarius* is an ectomycorrhizal genus [55] and it is possible that associations with specific host tree species help to explain high regionalism and habitat relationship. Several *Cortinarius* species described from Patagonia are thought to associate only with a specific *Nothofagaceae* host tree species, including, *C. magellanicus* Speg., *C. horakii* Valenz. & Esteve-Rav., *C. magellanicoalbus* Salgado Salomón & Peintner, *C. austronanceiensis* (Moser) Garnier, *C. capitellinus* Horak, *C. cinereus* Moser, *C. brachyspermus* Peintner & Moser, *C. glutinopallens* (Horak) Peintner & Moser, *C. cretaceus* (Horak) Horak and *C. roblerauli* Salgado Salomón & Peintner [1]. Due to undersampling and the difficult taxonomy of *Cortinarius* in Patagonia, it is possible that specific host associations could be more frequent than previously assumed. The newly described *C. pseudoxiphidipus* could represent one of these species with strong host preferences or host specificity since it was found only associated with *N. dombeyi*. Similarly, *C. egonii* and *C. gracilentus* showed specificity at the genus level and were only found with *Nothofagus* species. Within the genus *Cortinarius* there are also several ectomycorrhizal species with more generalist plant host associations, such as *C. albocanus* (Horak & Moser) Peintner & Moser, *C amoenus* (Moser & Horak) Garnier, *C. austroduracinus* Moser, *C. austrosalor* Moser, *C. collariatus* Horak & Moser and *C. flammuloides* Horak & Moser. They are all reportedly associated with *Nothofagaceae* species present in Patagonia [1]. We observed the same generalist pattern for *C. voluptatis* and *C. neuquensis*, which are associated with both *Lophozonia* and *Nothofagus* spp.

The phylogenetic placement of the five new species proposed in this work into subsections, sections, or even subgenera was not possible due to limited phylogenetic resolution and the lack of closely related reference taxa. The combined phylogenetic analysis of ITS, LSU and RPB1 resulted in a well-supported clade consisting of *C. pseudoxiphidipus*, *C. voluptatis* and *C. egonii.* They all have a similar habitus. However, we are reluctant to define new, possibly endemic South American sections of *Cortinarius* based only on three species. The lack of reference data shows that the South American *Cortinarius* diversity is still widely underexplored and it also suggests the presence of endemic *Cortinarius* lineages in this area. Up until now there is very little overlap between *Cortinarius* species described from Australia and New-Zealand [38] with the species reported from South American Nothofagaceae forests. Given the high diversity of Southern Hemisphere *Cortinarius* diversity and the lack of available reference sequences, it is not surprising that the phylogeny of *Cortinarius* species remains largely unresolved. Based on available data it seems likely that that endemic South American *Cortinarius* lineages exist. The recently proposed *Cortinarius* section *Austramericani* [16] (p. 1130) could be an example for such an endemic lineage of Patagonian *Cortinarius* species associated with *Nothofagus* species. More intense investigations on the diversity of this genus in South America are urgently needed, as this will not only clarify the Patagonian *Cortinarius* diversity, but will also provide fascinating insights into the evolution of ectomycorrhizal associations on a global scale. 

“Southern Gondwana” connections are often explained by the presence of specific host plants in the Southern Hemisphere that are absent in other regions and *vice versa* [56,57,58]. Due to their association with *Nothofagaceae* forests, Southern Gondwanan connections could also be assumed for several lineages of *Cortinarius*, as already proposed for other genera of ectomycorrhizal fungi [59,60,61,62]. For example, the /Pseudotriumphans clade of *Cortinarius* is shared between South America and Oceania (Australia-Tasmania and New Zealand), and therefore very likely to represent a Southern Hemisphere lineage of *Cortinarius* with wide *Nothofagaceae* host range [24] (p. 1049) and [23] (p. 1467). However, at the moment the available data only allow for speculation. A better knowledge of fungal diversity is needed to understand the evolutionary history of ectomycorrhizal fungi in Patagonian forests. 

The ITS region is frequently used for fungal species identification [37,63,64,65,66]. However, the ITS region has only minimal variation across *Cortinarius* and therefore probably underestimates the true diversity of this genus in natural ecosystems by up to 20% [67]. Barcoding is a powerful tool for ecological, environmental, or taxonomic research [68]. However, fungi occurring in the Southern Hemisphere are still largely under-represented in public databases [61], and data concerning their diversity and distribution are far from being complete, even when *Cortinarius* spp. are often quite dominant in studies of ectomycorrhizal fungi communities on roots and in soil [1,13,61,68,69]. This is especially true and important for fungal groups with immense species richness like *Cortinarius*, a widespread and important ectomycorrhizal genus from the South American *Nothofagaceae* forests with high ecological, forest restoration interests, and important non-timber forest products. 

## 5. Conclusions

The diversity of *Cortinarius* from the Northern Hemisphere and from New-Zealand and Australia have been extensively studied (e.g., [24,38,68,70,71,72]). However, aside from the pioneering work from Moser and Horak [5,26] there are relatively few studies that have focused on South American *Cortinarius* diversity [25]. Recent studies describing several new species or even new sections highlight this knowledge gap [8,12,15,16,67]. Based on our current knowledge, the *Cortinarius* species associated with *Nothofagaceae* species rarely or never occur outside the distribution range of their host trees [26,71,72,73,74,75], making it highly likely that, after losing the connection via Antarctica, endemic *Cortinarius* lineages evolved in South America and Australasia. *Cortinarius* harbors a high diversity in these habitats, including many taxa that are waiting to be discovered [15,16]. In the future, systematic approaches will be important to fully sample *Cortinarius* from South America. These approaches should include DNA barcording of South American *Cortinarius* herbarium and systematic sampling of *Nothofagaceae* forests by South American mycologists.

## Figures and Tables

**Table 1:** Material included in this study including ecological and habitat data, herbarium and GenBank/UNITE numbers.

Species	Site	Associated Species	GenBank/UNITE Number	Herbaria Number	Type	Sampling Date
*Cortinarius avellaneus*	Argentina, Neuquén, PNNH, Puerto Manzano	*Nothofagus dombeyi + N. pumilio*	UDB023898	IB 19630368	Type	18/4/1963
*Cortinarius caryotis*	Unknown	Unknown	KJ421039	F44422		Unknown
*Cortinarius caryotis*	New Zealand, Hawdon (Cass)	*Nothofagus* spp.	GU233348	PDD 71004	Holotype	21/4/1999
*Cortinarius caryotis*	New Zealand, UNP *, Lake Waikareiti Track	Unknown	GU233356	PDD 74305		11/5/2001
*Cortinarius cinereus*	Chile, Coyhaique	*Nothofagus dombeyi*	MH511098	CONCF0650		15/3/2007
*Cortinarius cinereus*	Argentina, Río Negro, PNNH **, Valle Frías	*Nothofagus dombeyi*	UDB023853	IB 19630117	Type	21/3/1963
*Cortinarius cinereus*	Chile	Unknown	UDB034963	IBFFG 650		Unknown
*Cortinarius dulcamarus*	New Zealand, North Canterbury, Medbury Scientific Reserve	*Kunzea ericoides*	MH101559	PDD 96951		26/5/2013
*Cortinarius dulcamarus*	New Zealand, Craigieburn	*Nothofagus* spp.	KJ635238	PDD 97534	Type	5/5/2009
*Cortinarius egonii*	Chile, Aysen, Carretera Austral, south of Bertrand port.	*Nothofagus pumilio* + *N. dombeyi*	MT925625	CT4418/FLAS-F-63487		3/5/2016
*Cortinarius egonii*	Argentina, Río Negro, PNNH, Steffen lake	*Nothofagus dombeyi*	MN707588	HCFC C246/IB 20170447		16/5/2017
*Cortinarius egonii*	Argentina, Río Negro, PNNH, Steffen lake	*Nothofagus dombeyi*	MN707589	HCFC C257/IB 20170257	Holotype/Isotype	16/5/2017
*Cortinarius egonii*	Argentina, Río Negro, PNNH, Steffen lake	*Nothofagus dombeyi*	MN707590	HCFC C258/IB 20170258		16/5/2017
*Cortinarius egonii*	Argentina, Chubut, PNLA ***, Colihual stream	*Nothofagus dombeyi*	MN707571, MW405257	HCFC C52/IB 20170324		11/4/2017
*Cortinarius egonii*	Argentina, Chubut, PNLA, Colihual stream	*Nothofagus dombeyi*	MN707574, MW405256, MW546832	HCFC C80/IB 20170342		18/4/2017
*Cortinarius egonii*	Argentina, Río Negro, Bariloche, Nahuel Huapi National Park, Los Rápidos	*Nothofagus dombeyi* + *N. antarctica*	MT925623	MES-1205 CORDC00006881		11/5/2016
*Cortinarius egonii*	Argentina, Río Negro, Bariloche, PNNH, Goye stream, near Colonia Suiza	*Nothofagus dombeyi* + *N. pumilio*	KY462608	MES-1888/CORDC00005629		12/5/2016
*Cortinarius egonii*	Argentina, Río Negro, Bariloche, PNNH, Los Rápidos	*Nothofagus antarctica*		MES-1930/CORDC00005614		13/5/2016
*Cortinarius egonii*	Argentina, Río Negro, Bariloche, PNNH, Road to Tronador	*Nothofagus pumilio*	MT925622	MES-2001 CORDC00005551		14/5/2016
*Cortinarius gracilentus*	Argentina, Chubut, PNLA, Camping area	*Nothofagus antarctica*	MN707580, MW405251	HCFC C171/IB 20170235		25/4/2017
*Cortinarius gracilentus*	Argentina, Chubut, PNLA, Rivadavia river	*Nothofagus dombeyi*	MN707572	HCFC C66/IB 20170334	Holotype/Isotype	18/4/2017
*Cortinarius ‘gymnopiloides’*	New Zealand	Unknown	AF389147	ZT NZ68501	Type	Unknown
*Cortinarius macilentus*	Argentina, Río Negro, PNNH, Valle Frías	*Nothofagus dombeyi*	UDB023869	IB19630184	Type	24/3/1963
*Cortinarius micaceus*	Argentina, Río Negro, PNNH, Valle Frías	*Nothofagus dombeyi + N. antarctica + N. pumilio*	UDB023868	IB 19630182	Type	20/3/1963
*Cortinarius mitis*	Argentina, Río Negro, PNNH, Valle Frías	*Nothofagus dombeyi*	UDB023858	IB19630139	Type	22/3/1963
*Cortinarius neuquensis*	Argentina, Bariloche, PNNH, along road halfway to Tronador.	*Nothofagus antarctica*	MT925952	MES-1148 CORDC00005190		9/5/2015
*Cortinarius neuquensis*	Argentina, Neuquén, PNL ****, Ñorquinco Lake	*Lophozonia alpina+ L. obliqua*	MN707581	HCFC C196 IB 20170218		3/5/2017
*Cortinarius neuquensis*	Argentina, Neuquén, Chañy Protected Area, Chañy stream	*Nothofagus antarctica + A. araucana*	MN707582	HCFC C206 IB 20170222	Holotype/Isotype	4/5/2017
*Cortinarius neuquensis*	Argentina, Neuquén, Chañy Protected Area, Chañy stream	*N. antarctica + A. araucana*	MN707583, MW405255, MW546831	HCFC C210 IB 20170224		4/5/2017
*Cortinarius neuquensis*	Chile, Osorno, PNP *****, last stop near Aguas Calientes	*Nothofagus dombeyi*	MT925953	MES-1551 FLAS-F-64363		3/5/2016
*Cortinarius neuquensis*	Chile, Osorno, PNP, foothills of Volcan Puyehue, up the road past El Caulle north of Rio Golgol	*Nothofagus dombeyi*	KY462509	MES-1638 FLAS-F-64429		4/5/2016
*Cortinarius neuquensis*	Argentina, Bariloche, PNNH, Road to Tronador	*Nothofagus antarctica*	MT925951	MES-2009 CORDC00005547		14/5/2016
*Cortinarius neuquensis*	Chile	*Lophozonia alpina*	KY462703	MES-988 FLAS-F-63016		Unknown
*Cortinarius nitellinus*	Argentina, Neuquén, PNNH, Puerto Manzano	*Nothofagus dombeyi*	UDB023833	IB19630044	Type	12/3/1963
*Cortinarius ‘perelegans’*	New Zealand	Unknown	JX178615	OTA 60285		Unknown
*Cortinarius pseudoxiphidipus*	Argentina, Chubut, PNLA, Rivadavia river	*Nothofagus dombeyi*	MN707573, MW405254, MW546828	HCFC C78 IB 20170340		18/4/2017
*Cortinarius pseudoxiphidipus*	Argentina, Chubut, PNLA, Rivadavia river	*Nothofagus dombeyi*	MN707575, MW405252	HCFC C88 IB 20170347	Holotype/Isotype	18/4/2017
*Cortinarius pseudoxiphidipus*	Argentina, Chubut, PNLA, Rivadavia river	*Nothofagus dombeyi*	MN707576, MW405253, MW546829	HCFC C90 IB 20170441		18/4/2017
*Cortinarius rhodophyllus*	Unknown	Unknown	KJ421051	TUB 020416		Unknown
*Cortinarius rufus*	Argentina, Neuquén, PNNH, Puerto Manzano	*N. pumilio*	MF568564	IB19630369	Type	18/4/1963
*Cortinarius rufus*	Argentina, Río Negro, PNNH, Arroyo Goye near Colonia Suiza	*Nothofagus dombeyi + N. pumilio*	MF568565	K(M)234990		12/5/2016
*Cortinarius semiamictus*	Argentina, Río Negro, Paso de las Nubes, Frías Valley	*Nothofagus dombeyi + N. antarctica*	UDB023828	IB 19620161	Type	7/4/1962
*Cortinarius* sp.	Australia	Unknown	MG553066	PERTH:06435416 FC393		Unknown
*Cortinarius* sp.	Chile, Osorno, PNP, foothills of Volcan Puyehue, up the road past El Caulle north of Rio Golgol	*Nothofagus dombeyi*	KY462487	MES-1597 FLAS-F-64397		Unknown
*Cortinarius* sp.	Chile, Osorno, PNP, foothills of Volcan Puyehue, up the road past El Caulle north of Rio Golgol	*Nothofagus dombeyi*	KY462492	MES-1602 FLAS-F-64401		Unknown
*Cortinarius* sp.	Chile, Osorno, PNP, foothills of Volcan Puyehue, up the road past El Caulle north of Rio Golgol	*Nothofagus dombeyi*	KY462567	MES-1801 FLAS-F-64558		Unknown
*Cortinarius* sp.	Argentina, Nahuel Huapi National Park, Arroyo Goye, near Colonia Suiza	Unknown	KY462598	MES-1859 CORDC00005597		Unknown
*Cortinarius subrufus*	Argentina, Río Negro, PNNH, Hess lake	*Nothofagus antarctica*	MF568560	K(M)235093		17/5/2016
*Cortinarius subrufus*	Chile, Magallanes, Karukinka Reserve, Vicuña station	*Nothofagus antarctica*	MF568561	K(M)235583		27/3/2017
*Cortinarius subrufus*	Chile, Magallanes, Karukinka Reserve, Vicuña station	*Nothofagus antarctica*	MF568562	K(M)235584		26/3/2017
*Cortinarius verniciorum*	New Zealand, Fiordland, Te Anau Downs Motel	*Leptospermum*, possibly *Nothofagus*	JQ287679	PDD 94010		25/4/2008
*Cortinarius verniciorum*	New Zealand, Fiordland, Te Anau Downs Motel	*Leptospermum*, possibly *Nothofagus*	NR157876	PDD 94010	Type	25/4/2008
*Cortinarius viscilaetus*	New Zealand, Totara, Milford Road	*Nothofagus* spp.	KT875206	PDD 107734		18/5/2015
*Cortinarius viscilaetus*	New Zealand, Te Anau, Kepler Track	*Nothofagus* spp.	GU233353	PDD 71010	Type	18/4/1997
*Cortinarius voluptatis*	Argentina, Chubut, PNLA, Rivadavia Camping area	*Nothofagus antarctica*	MN707579	CIEFAP157/IB 20170109		25/4/2017
*Cortinarius voluptatis*	Argentina, Neuquén, PNL, Yuco region	*Lophozonia alpina + L. obliqua*	MN707584, MW405260	HCFC C218/IB 20170229		5/5/2017
*Cortinarius voluptatis*	Argentina, Neuquén, PNL, Yuco region	*Lophozonia alpina + L. obliqua*	MN707585, MW405258, MW546830	HCFC C219/IB 20170231	Holotype/Isotype	5/5/2017
*Cortinarius voluptatis*	Argentina, Neuquén, PNL, Yuco region	*Lophozonia alpina + L. obliqua*	MN707587, MW405259	HCFC C230/IB 20170238		5/5/2017
Uncultured *Cortinarius*	Argentina, Neuquén, PNL, Yuco region	*Lophozonia alpina*	KJ701302	Environmental		Unknown
Uncultured fungus	Argentina	*Nothofagus pumilio*	JX316449/UDB008462	Environmental		Unknown
Uncultured fungus	Argentina	*Lophozonia alpina*	JX316363	Environmental		Unknown

UNP *: Urewera National Park, PNNH **: Nahuel Huapi National Park, PNLA *** Los Alerces National Park, PNL ****: Lanín National Park, PNP *****: Puyehue National Park.

## Data Availability

Data is contained within the article or supplementary material.

## Supplementary Materials

The following are available online at https://www.mdpi.com/article/10.3390/life11050420/s1, Figure S1: Phylogenetic relationship (Bayesian consensus tree) of four new species of *Cortinarius* from South American *Nothofagaceae* forests, based on combined rDNA ITS-LSU and RPB1 sequences. Bayesian Posterior Probabilities are provided beside nodes. *C. gracilentus* was not included in the analysis. Sequences generated from the new species presented are highlighted in red.
