# Supplementary figures and images for: Loose Ends in the Cortinarius Phylogeny: Five New Myxotelamonoid Species Indicate a High Diversity of These Ectomycorrhizal Fungi with South American Nothofagaceae
Source: Life (Basel). 2021 May 5;11(5):420. doi: 10.3390/life11050420 (PMC8148173; doi:10.3390/life11050420)

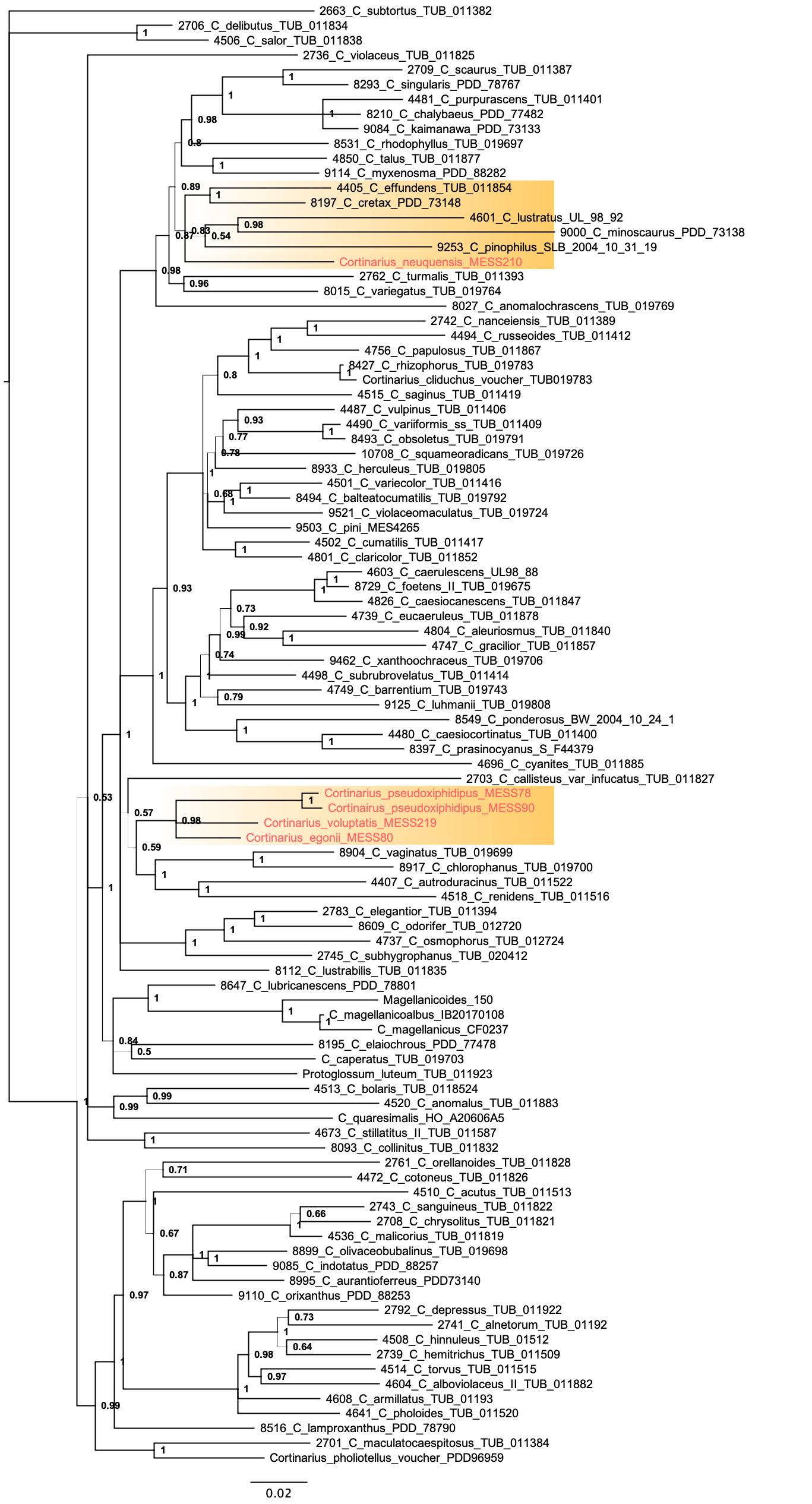

Supplement: Supplementary file 1 [file life-11-00420-s001.zip › life-1187518-supplementary.jpg]
